# Intensive Patient Education Improves Glycaemic Control in Diabetes Compared to Conventional Education: A Randomised Controlled Trial in a Nigerian Tertiary Care Hospital

**DOI:** 10.1371/journal.pone.0168835

**Published:** 2017-01-03

**Authors:** Okon Essien, Akaninyene Otu, Victor Umoh, Ofem Enang, Joseph Paul Hicks, John Walley

**Affiliations:** 1 Department of Internal Medicine, University of Calabar, Calabar, Cross River State, Nigeria; 2 Department of Medicine, University of Uyo, Uyo, Akwa Ibom State, Nigeria; 3 Nuffield Centre for International Health and Development, Leeds Institute of Health Sciences, University of Leeds, Leeds, United Kingdom; Weill Cornell Medical College Qatar, QATAR

## Abstract

**Background:**

Diabetes is now a global epidemic, but most cases are now in low- and middle-income countries. Diabetes self-management education (DSME) is key to enabling patients to manage their chronic condition and can reduce the occurrence of costly and devastating complications. However, there is limited evidence on the effectiveness of different DSME programmes in resource limited settings.

**Methods:**

We conducted an unblinded, parallel-group, individually-randomised controlled trial at the University of Calabar Teaching Hospital (Nigeria) to evaluate whether an intensive and systematic DSME programme, using structured guidelines, improved glycaemic control compared to the existing ad hoc patient education (clinical practice was unchanged). Eligible patients (≥18 years, HbA_1c_ > 8.5% and physically able to participate) were randomly allocated by permuted block randomisation to participate for six months in either an intensive or conventional education group. The primary outcome was HbA_1c_ (%) at six-months.

**Results:**

We randomised 59 participants to each group and obtained six-month HbA1c outcomes from 53 and 51 participants in the intensive and conventional education groups, respectively. Intensive group participants had a mean six-month HbA_1c_ (%) of 8.4 (95% CI: 8 to 8.9), while participants in the conventional education group had a mean six-month HbA_1c_ (%) of 10.2 (95% CI: 9.8 to 10.7). The difference was statistically (P < 0.0001) and clinically significant, with intensive group participants having HbA_1c_ outcomes on average -1.8 (95% CI: -2.4 to -1.2) percentage points lower than conventional group participants. Results were robust to adjustment for a range of covariates and multiple imputation of missing outcome data.

**Conclusions:**

This study demonstrates the effectiveness of a structured, guideline-based DSME intervention in a LMIC setting versus a pragmatic comparator. The intervention is potentially replicable at other levels of the Nigerian healthcare system and in other LMICs, where nurses/diabetes educators can run the programme.

**Trial Registration:**

Pan African Clinical Trial Registry PACTR20130200047835

## Introduction

It is now estimated that between 340 and 536 million adults (aged 20–79 years) worldwide have diabetes, with the disease accounting for an estimated 14.5% of global all-cause mortality in 2015 [1]. However, over the past decade the prevalence of diabetes has risen most rapidly in low- and middle-income countries (LMICs) [2], where it is now estimated that around 75% of people with diabetes live [1]. Low- and middle-income countries also generally lack the resources with which to adequately deal with this growing diabetes epidemic. For example, in 2015 it is estimated that just 19% of global health expenditure on diabetes occurred in LMICs [1].

Poorly managed diabetes results in hyperglycaemia and eventually serious microvascular and macrovascular complications, which can lead to cardiovascular events, kidney failure, blindness, nerve damage and peripheral arterial disease, ultimately resulting in disability and/or mortality [3]. However, tightly controlling blood glucose levels through the effective use of medication and the management of key lifestyle factors, such as diet and exercise, reduces the risk of serious complications developing and progressing [4, 5]. Given the chronic nature of the condition, it is therefore vital that individuals develop the knowledge and skills necessary to effectively manage their condition on a day-to-day basis away from professional healthcare facilities, and thereby prevent or delay the development of complications [6]. Diabetes self-management education (DSME) is therefore seen as “a critical element of care for all people with diabetes and those at risk of developing the disease [6].” However, in LMICs DSME is rarely available or is of poor quality [7], and unlike in many high-income countries [6] context specific guidelines rarely exist for clinical staff to utilise.

A number of meta-analyses of randomised controlled trials (RCTs) have provided support for DSME, demonstrating that compared to routine treatment DSME programmes typically lead to small but significant improvements in blood glucose control [8–11]. Both group-based and individual-based DSME programmes appear effective [8–11], but group-based approaches are typically cheaper, and offer the advantage of allowing patients to meet and discuss their issues together. Unsurprisingly, very few RCTs have evaluated DSME programmes in LMICs (but see [12–15]), and in LMICs people with diabetes typically lack the required skills and knowledge to effectively self-manage their condition [16, 17]. For example, in the Nigerian health system there has been a long-term systematic failure to adequately control blood glucose levels in most diabetes patients [18]. Furthermore, DSME is also only available on a limited basis, health workers often lack adequate knowledge about how to effectively manage patients with diabetes, and the majority of health care facilities do not employ protocol-driven care of patients [19]. Consequently, education of patients and health workers is seen as key to improving this dire situation [18].

Therefore, we developed an intensive, structured and systematic group-based DSME programme that could be delivered by nurses or doctors to people with either type 1 or type 2 diabetes, and was tailored to the Nigerian context. We hypothesised that our intensive group-based DSME would be more effective at helping diabetes patients successfully manage their blood glucose levels compared to the existing unstructured, non-systematic and ad hoc group-based DSME available within a tertiary hospital. This was therefore a more pragmatic and useful comparison than to no DSME.

## Methods

For the trial protocol see S1 Text Trial Protocol. The trial was reported according to CONSORT standards: see S2 Text CONSORT Checklist and S1 Table CONSORT Extension for Abstracts Checklist.

### Study design, setting and patients

The trial had a parallel-group, individually-randomised, controlled design, and evaluated whether the intensive, structured and systematic education programme was superior to conventional education. Participants were recruited between 1/09/2013 and 31/05/2014 from new and existing type 1 and type 2 diabetes patients attending the endocrinology clinic at the University of Calabar Teaching Hospital (UCTH), located in Cross River State, Nigeria. Within Calabar city the prevalence of undiagnosed diabetes is estimated at 7% [20], while the national prevalence of diabetes within Nigeria is tentatively estimated at around 5% [1]. Due to a delay in the release of funds by the organisations that supported this research, there was a delay between trial registration and commencement of recruitment of participants.

Individuals were considered eligible to participate in the study if they were 1) diagnosed with either type 1 or type 2 diabetes, 2) had glycosylated haemoglobin (HbA_1c_) levels >8.5% when measured at a maximum of 30 days prior to randomisation, 3) were aged 18 years or older, 4) were able to engage in moderate exercise without issue and 5) were free of any eye disease that would otherwise limit their ability to read printed materials. All eligible patients were given a full explanation of the study by a research assistant and invited to participate, with written informed consent required prior to participation. Those providing consent then had their baseline HbA_1c_ measured by a nurse, who was not subsequently involved in patient education within either arm, using a Clover A1c Analyser^™^ (EuroMedix), based on a 4μl sample of capillary blood from a finger prick. Additional clinical and sociodemographic baseline data were also collected by the research assistant.

### Randomisation and blinding

Following baseline data collection participants were then randomly allocated in a 1:1 ratio to either receive the intensive education programme or conventional education. Randomisation was carried out by the research assistant using randomisation lists produced by an independent statistician, which were kept in sealed, opaque envelopes until required. Randomisation was based on a permuted block design, calculated using a computer program, with a fixed block size of 4. Due to the design of the trial it was not possible to blind participants, and it was also not possible to blind the doctors and nurses running the education sessions in either arm, but nurses recording participants’ follow-up outcome data were blinded to participant allocation.

### Processes

The aim was to develop a comprehensive DSME programme built from different evidence-based components, which could be delivered by nurses or doctors to groups of diabetes patients, and which could be easily scaled-up and implemented in Nigeria primary and secondary care facilities (and was therefore also applicable to other similar LMIC healthcare contexts). The education programme was also designed to be applicable to patients with either type 1 or type 2 diabetes, with lifestyle education applicable to both forms of diabetes, but medication-related education specific to both types. These aims were achieved by tailoring the Health Educator Desk Guide of the COMDIS-HSD group in the United Kingdom [21], which reflects the core educational elements recommended by the International Diabetes Federation (IDF) [22], to the Nigerian healthcare context.

No changes were made during the trial to the clinical treatment provided by doctors to diabetes patients in either group. Therefore, the only difference for diabetes patients in each group was in terms of the form of DSME they received. Participants randomised to the intensive education arm were invited and encouraged by clinical staff to attend a total of 12 structured teaching sessions lasting around two hours each, which they attended fortnightly over a six month period. On average there were six to eight participants in a session. The initial six sessions were delivered by three doctors, while the final six sessions were delivered by three nurses. All doctors and nurses involved in running the intensive education sessions were from the endocrinology clinic of the UCTH, and all had previously (and separately to this study) been trained as certified diabetes educators via a training programme run by the IDF and supported by a number of pharmaceutical companies. In addition, prior to the start of the study they were also trained on how to use education desk guide during the sessions by a team of researchers comprising AO, OEs and OEn.

Each education session was comprised of lectures and group discussions centred on one of the core educational elements recommended by the IDF [22], as reflected in the COMDIS-HSD Health Educator Desk Guide [21] and tailored to the Nigerian context for this study. Themes were systematically covered across the sessions and included 1) diet and nutrition, 2) compliance with medications and the mechanism of medication action (covering medications for both type 1 and 2 diabetes), 3) exercise, 4) foot and skin care, 5) self-monitoring of glucose levels (although as per usual care, i.e. that received by conventional group participants, glucometers were not provided to participants, but they were encouraged to purchase one), 6) smoking cessation and 7) blood pressure and cholesterol monitoring. The sessions were interactive and included generic diabetes education videos, and generic diabetes education leaflets were also provided for participants to take home to reinforce the desired lifestyle modifications.

Attendance to these intervention sessions was assessed and found to be very high (>95%). No further education was given outside the twelve educational sessions. Care was taken to provide the intensive education sessions at a location that was well removed from the endocrinology clinic and sessions were also held separately to participants’ clinical appointments (with participants going home after sessions) to limit the risk of interactions between participants in the different study groups. Mobile phone messages were also used regularly to remind participants to attend the educational sessions, to limit the losses to follow up. There was no tailoring or modification of the intervention throughout the course of the trial.

The DSME available to participants in the conventional group was unchanged from that offered to all patients with diabetes in the endocrinology clinic under usual care. Consequently, the DSME sessions were not mandatory, but participants were encouraged to attend by clinical staff (with attendance typically being high in usual care). Sessions were held before the start of participants’ clinical visits, which typically occurred (unless exhibiting complications) once every three to four weeks. Therefore, most conventional group participants attended around six DSME sessions in total during the trial. In each session there were usually around three to four participants as well as any non-trial diabetes patients attending their routine appointments, with sessions held in a room separate to the clinical area. Following the sessions participants then attended their clinical consultations with their doctors.

These sessions were also run by three nurses and three doctors who had all previously been trained as certified diabetes educators (via the same process as those educators running the intensive DSME sessions), but these were different staff to those running the intensive education sessions. Sessions typically consisted of a non-interactive lecture lasting around 30 to 45 minutes, with around two-thirds of the information provided by nurses and the remaining third by doctors. Principles of diabetes care such as diet, nutrition and exercise, compliance with medications, and foot and skin care were presented in a didactic way during sessions. However, there was no structured or systematic set of themes to be covered, sessions were not based on any specific guidelines and no videos or educational materials were provided. There was also no use of mobile phone reminder messages. Trial participants’ attendance at the conventional educational sessions was also found to be very high (>95%).

### Outcomes and follow-up

The primary outcome was HbA_1c_ (%), recorded six months after randomisation (± at most five days). There were no secondary outcomes. HbA_1c_ measurements were taken by nurses, using the same methods as those used at baseline. Nurses were not involved in delivering participant education in either group, and were blinded to participants’ allocation. The trial ended once all participants not lost to follow-up had their six-month HbA_1c_ measurements taken.

### Statistical analysis

It was estimated that 118 participants (59 per arm) were required to detect a difference of at least 0.7 percentage points in mean HbA_1c_ (%) between treatment groups with 80% power, assuming a common standard deviation of 1.35, using a two-sided test and a significance (α) level of 0.05.

In all analyses participants were analysed according to the treatment group they were originally assigned to. Outcome data were missing for 14 patients, but an exploration of this missing data indicated no clear patterns to the missingness. Therefore, the primary analysis assumed missing data were missing completely at random, and used multivariate linear regression of complete cases to compare participants’ HbA_1c_ at follow-up between the intensive and conventional education groups, after adjusting for baseline HbA_1c_ levels. To evaluate the robustness of these ‘crude’ results to any imbalances in influential baseline characteristics an adjusted analysis of complete cases was also conducted that controlled for age, sex, diabetes type, education level (none/primary, secondary or tertiary) and years since diabetes diagnosis, in addition to baseline HbA_1c_ levels (type of diabetes medication was not adjusted for because it reflected participants’ diabetes type in all but one case). Similarly, to test the sensitivity of the results from the complete case analyses to a missing at random assumption the crude and adjusted analyses described above were repeated with missing outcome data imputed via a multiple imputation process (see S3 Text for details). All analyses were conducted using SAS (SAS 9.3, SAS Institute Inc), with two-sided hypothesis testing and significance at the 0.05 level.

### Ethics

The study received full ethical approval from the Health Research and Ethics Committee of the UCTH, and was registered with the Pan African Clinical Trial Registry (unique registry identification number: PACTR201302000478315.

## Results

**Fig 1:**
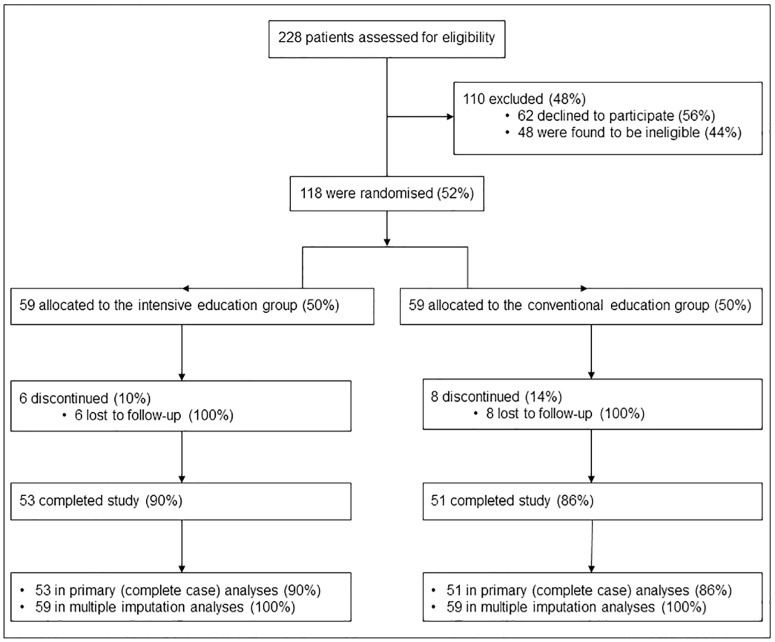
CONSORT recruitment and trial participant flow.

A total of 228 individuals were screened and 48 were found to be ineligible (Fig 1). All eligible participants were invited to join the trial. Sixty-two declined to participate, but the remaining 118 eligible individuals provided written informed consent and were randomised equally between treatment groups following collection of baseline data (Fig 1). A total of 14 (11.9%) participants, 6 (10.2%) from the intensive education group and 8 (13.6%) from the conventional education group, were lost to follow-up (Fig 1) after all became uncontactable during the trial, and therefore did not provide outcome data. Outcome data were available for all remaining participants who completed the study: 53 (90%) in the intensive education group and 51 (86%) in the conventional education group (Fig 1).

Most participants had type 2 diabetes (85.6%), and were taking oral hypoglycaemic drugs (84.7%) rather than insulin or insulin plus oral hypoglycaemic drugs (14.4%), reflecting the distribution of type 1 and type 2 diabetes patients in the trial population (Table 1). Participants had typically been diagnosed as having diabetes for a number of years (mean 6.5 years), and most were middle-aged (IQR: 48–58.8), clinically overweight (79.7% had a BMI ≥25), and 70.3% had secondary or tertiary education (Table 1). There was some indication of potentially important imbalances in sex, and to a lesser extent education and type of diabetes, but baseline characteristics appeared otherwise well balanced (Table 1).

**Table 1:** Baseline demographic and clinical characteristics by treatment group and overall.

		Intensive education (N = 59)	Conventional education (N = 59)	All participants (N = 118)
**Age**		52.6 (10.9)	52.8 (10.1)	52.7 (10.5)
**Sex**				
	Male	28 (47.5)	19 (32.2)	47 (79.7)
	Female	31 (52.5)	40 (67.8)	71 (60.2)
**Education level**				
	None/Primary	14 (23.7)	18 (30.5)	32 (54.2)
	Secondary	21 (35.6)	18 (30.5)	39 (66.1)
	Tertiary	24 (40.7)	23 (39)	47 (79.7)
**Body mass index, mean (SD)**^a^		29.6 (8.8)	28.3 (5.8)	28.9 (7.5)
**Waist circumference (cm)**		96.8 (9.1)	95.3 (8.8)	96.1 (8.9)
**Years since diabetes diagnosis**		6.9 (4.7)	6.1 (3.8)	6.5 (4.3)
**Diabetes type**				
	Type 1	10 (16.9)	7 (11.9)	17 (14.4)
	Type 2	49 (83)	52 (88.1)	101 (85.6)
**Medication**				
	Diet only	0 (0)	1 (1.7)	1 (0.8)
	Oral hypoglycaemic drugs	49 (83)	51 (86.4)	100 (84.7)
	Insulin or insulin plus oral hypoglycaemic drugs	10 (16.9)	7 (11.9)	17 (14.4)
**Baseline HbA**_**1c**_ **%**		10.9 (1.7)	10.5 (1.5)	10.7 (1.6)

Values are n (%) for categorical variables or mean (SD).

^a^ Body mass index = weight (kg) / height (m^2^).

The primary analysis indicated that participants in the intensive education group had significantly lower HbA_1c_ levels at six-month follow-up compared to participants in the conventional education group, with a mean estimated difference of -1.8 (95% CI: -2.4 to -1.2) in HbA_1c_ percentage points (Table 2). This result appeared robust, with no substantive difference found in the results from the adjusted analysis and the analyses using multiply imputed missing data (Table 2 & S3 Text).

**Table 2:** Multiple regression results for HbA_1c_ outcomes at six-month follow-up for participants in the intensive and conventional education groups.

	Intensive education group	Conventional education group	Intensive-conventional group difference	P-value
**N**	53/59	51/59	104/118	
**Crude HbA**_**1c**_ **%**^a^	8.4 (8 to 8.9)	10.2 (9.8 to 10.7)	-1.8 (-2.4 to -1.2)	<0.0001
**Fully adjusted HbA**_**1c**_ **%**^b^	8.3 (7.8 to 8.7)	10.1 (9.5 to 10.7)	-1.8 (-2.4 to -1.2)	<0.0001

Group outcomes are least-squares means (95% confidence interval) and group differences are multiple regression group dummy-variable coefficients (95% confidence interval). N is the number of participants with outcome measures recorded (complete cases) / the number of participants randomised.

^a^Based on a multivariate linear regression model of complete cases, only adjusting for baseline HbA_1c_ (%).

^b^Based on a multivariate linear regression model of complete cases, adjusting for baseline HbA_1c_ (%), age, sex, diabetes type, education level and years since diabetes diagnosis.

## Discussion

This study demonstrates that an intensive, structured and systematic group-based DSME programme can help patients manage their blood glucose levels more effectively than conventional, ad hoc group-based DSME within a LMIC healthcare setting. Specifically, we found robust results demonstrating that patients in the intensive education group had HbA_1c_ (%) values at six-month follow-up that were on average -1.8 (95% CI: -2.4 to -1.2) percentage points lower than patients in the conventional education group. This highly statistically significant difference is clinically very meaningful. For example, the UK Prospective Diabetes Study showed that the risk of diabetes complications drops by 35% for every percentage point decrease in HbA_1c_ [23].

To the best of our knowledge this is the first RCT of a DSME intervention in Nigeria, although a five-year non-randomised study in another Nigerian tertiary hospital compared treatment involving longer doctor-patient consultations and participation in ‘health education forums’ to routine treatment, and provided some evidence of improved blood glucose control and reduced occurrence of morbidity [24]. However, a small number of RCTs evaluating DSME interventions for people with diabetes have been conducted in other LMICs, including Costa Rica, China and Thailand, and have also demonstrated clinically significant improvements in biomedical outcomes [12, 14, 15]. Conversely, an RCT of a DSME programme in under-served communities in South Africa produced no positive results relating to biomedical outcomes, but a process evaluation indicated serious problems with the running of the programme and with patient attendance [13]. Trials of similar diabetes (and CVD) educational guidelines as used here, along with clinical guidelines, are also on-going in Swaziland, Pakistan and China by the COMDIS-HSD group, and the guides, training modules and tools are all freely available online for adaptation in other LMICs (http://comdis-hsd.leeds.ac.uk/resources/technical-guidelines/).

Diabetes self-management education programmes are seen as vital and fundamental tools for enabling patients to effectively manage their diabetes in high-income countries [6]. Therefore, the results presented here, and those from similar trials in LMICs discussed above, indicate that (when properly functioning) DSME programmes can lead to clinically important improvements in patients’ management of their diabetes in LMIC settings. This is an important finding given that the burden from the growing global epidemic of diabetes is felt overwhelmingly in LMICs such as Nigeria [1]. Clearly there appears to be a small but growing base of evidence supporting the roll-out of such programmes in LMIC healthcare settings. Specifically in Nigeria there appears a need for such programmes within general hospitals, as well as both public and private primary care centres, and following the experience in this study the National Council of Health plans to adopt the educational guidelines and roll them out nationally.

This study was conducted in a busy tertiary hospital in Nigeria and pragmatically compared the existing conventional DSME available to patients (representing a best-case scenario for diabetes patients in Nigeria), which involved ad hoc, unstructured and non-systematic didactic provision of information, with a structured, systematic and intensive DSME programme. This programme was based on structured guideline counselling, and the guidelines were designed to be suitable for nurses, educators or doctors in more peripheral hospitals and health centres, and should therefore be replicable in similar settings. Generally, diabetes care in Nigeria is almost solely provided by medical doctors who educate their patients based on their individual experiences and understanding of diabetes care, which can be poor [25, 26]. There are also less than 30,000 doctors in Nigeria today [27], and the approximate average doctor:patient ratio of 1:53,333 indicates that their time should not be spent on educational programmes when such roles may be taken up by nurses or specialist educators. Such a situation is typical for LMICs. Therefore, a nurse- or specialist educator-led approach has the potential to reduce the burden of care for doctors, while being replicable in the primary and secondary levels of care in LMIC health settings where doctors are scarce and their time spread thinly. Clearly though, a major barrier (probably the biggest) to implementing effective DSME programmes in Nigeria and other LMICs is the issue of cost and resources. For example, whilst Nigerian nurses and dieticians overwhelmingly support the introduction of DSME programmes, believing them to be of benefit for their patients, only around one-third believe their establishments would be currently capable of implementing them [28].

The study had a number of limitations. First, the initial six intensive education sessions were led by doctors, when ideally all sessions would have been led by nurses to maximise the generalisability of the results to LMIC settings where nurses or educators would be the most obvious group to run such programmes. However, the education sessions were designed to be simple to run, and we encountered nothing to suggest that nurses, who successfully ran the latter six sessions, would not be able to successfully run the entire programme. Second, the trial only had a six-month follow-up period, and it is not clear how sustainable patient adherence and improvements in biomedical outcomes would be over longer timescales. Third, the mechanisms by which the programme achieved its effects were not explicitly investigated. These two limitations are also shared by the other RCTs of DSME programmes run in LMICs discussed previously [12, 14, 15], where follow-up periods ranged from three to six months, and the mechanisms by which these interventions worked were not explicitly investigated (although the South African trial, which did not produce positive results, did conduct a process evaluation [13]).

Four, it is unclear how generalisable the results are because the patient population and setting of the study do not closely represent the typical Nigerian diabetes patient and health care context. Only 2.5% of participants had no education and 40% had tertiary education, whilst the estimated rate of illiteracy in Nigeria is 40.4% in individuals aged 15 or more [29]. Although the educational materials used during the trial were simplified and relied largely on pictorial representations to drive home key messages, participants with higher levels of education may have benefitted more from the written, and indeed oral, provision of information. It is therefore unclear to what extent the atypically high level of education among participants may have been an enabling factor in the success of the trial. For example, studies have demonstrated positive relationships between the education levels of Nigerian diabetes patients’ and their knowledge about diabetes and its management, and the appropriate use of insulin [16, 17]. The trial was also conducted in just one public teaching hospital, and teaching hospitals are generally better equipped and staffed than either public general hospitals or most private hospitals, as they are directly funded by the federal government. Finally, given the unblinded nature of the study, common to many complex interventions, there is a possibility that trial effects, such as the Hawthorne effect, may have biased the estimated treatment effect of the intervention [30].

In conclusion, this study indicates that an intensive, structured and systematic guideline-based DSME intervention can improve biomedical outcomes in diabetes patients compared to unstructured, non-systematic and ad hoc DSME over relatively short timescales, a finding supported by a small number of similar RCTs in LMIC settings. Combining this structured education, led by nurses or specialist educators, together with quality clinical case management has the potential to improve diabetes management in LMICs, and thereby reduce the frequency of complications and their associated and catastrophic human and financial costs. Future studies of DSME programmes in LMICs should explore the following two sets of key questions. First, how feasible, cost-effective and generalisable are DSME programmes in LMICs? Second, how sustainable are the effects of such programmes in LMIC settings, and by what mechanisms do they generate their effects?

## Supporting Information

S1 Text Trial Protocol. (DOCX)

S2 Text CONSORT Checklist. (DOC)

S3 Text Multiple Imputation Details. (DOCX)

S1 Table CONSORT Extension for Abstracts Checklist. (DOC)

S2 Table Multiple regression results for HbA1c outcomes at six-month follow-up for participants in the intensive and conventional education groups with missing data multiply imputed. (DOCX)

S3 Table Trial Data. (XLSX)

S4 Table TIDieR checklist. (DOCX)
