# Supplementary material for: Intensive Patient Education Improves Glycaemic Control in Diabetes Compared to Conventional Education: A Randomised Controlled Trial in a Nigerian Tertiary Care Hospital
Source: PLoS One. 2017 Jan 3;12(1):e0168835. doi: 10.1371/journal.pone.0168835 (PMC5207750; doi:10.1371/journal.pone.0168835)
Supplement: S1 Text — (DOCX) [file pone.0168835.s001.docx]

**STUDY PROTOCOL**

Title: Intensive Patient Education Improves Glycaemic Control in Diabetes Compared to Conventional Education: a Randomised Controlled Trial in a Nigerian Tertiary Care Hospital

**Researchers**

Okon Essien^1^, Akaninyene Otu^1^*, Victor Umoh^2^ , Ofem Enang^1^ , Joseph Paul Hicks^3^, John Walley^3^

^1^Department of Internal Medicine

University of Calabar

Calabar

Cross River State

Nigeria

2Department of Medicine

University of Uyo

Uyo

Akwa Ibom State

Nigeria

3 Nuffield Centre for International Health and Development

Leeds Institute of Health Sciences

University of Leeds

Leeds

United Kingdom

### Introduction

Diabetes mellitus (DM) is a chronic disease that is very costly to manage and is associated with very serious comorbidities.^1^ Globally, the increase in diabetes prevalence that has not been limited to high-income countries but has involved low and middle income countries (LMICs) as well. Recent estimates from the International Diabetes Federation (IDF) put the global burden of diabetes mellitus at 382 million with 80% of them residing in LMICs.^2^ Studies have shown that the occurrence of microvascular and macrovascular complications of diabetes can be reduced by strict glycaemic control.^3-5^ Glycaemic control can now be monitored by measuring glycosylated haemoglobin (HbA_1c_) levels which has been accepted as a reliable and primary index of glycaemia since the mid-1970s.^6^ Achieving good glycaemic control requires the commitment of patients with diabetes to improving their circumstances with support from health workers. Education has long been considered the cornerstone of diabetes care as it provides patients with the appropriate skills and knowledge thereby causing them to assume greater responsibility for their care.^7^ It provides the foundation upon which other vital factors such as nutrition, medication, lifestyle and behavioural modifications are built upon. However, it is estimated that as many as 50-80% of diabetes patients lack the knowledge and skills to effectively manage their condition.^8^

There is no consensus on the most efficient way of delivering education to persons with diabetes. In the endocrinology clinic of the University of Calabar Teaching Hospital (UCTH) Nigeria, diabetes education is routinely given in the course of caring for patients with diabetes. This is usually provided by diabetes nurse educators and reinforced by medical doctors during their interaction with patients in the consulting clinics. We hypothesized that an intensive education programme provided in small groups and reinforced by educational leaflets would be more effective than conventional diabetes education in reducing HbA_1c_ levels when provided over a six month period. Thus, this study compared the effectiveness of an intensive group educational programme with the conventional educational programme for eligible patients with diabetes attending the endocrinology clinic in the UCTH.

### Objective

This study aims to evaluate the effectiveness of an intensive education programme designed to help diabetes patients in University of Calabar Teaching Hospital manage their condition and improve glycosylated haemoglobin (HbA_1c_) levels.

**Methods**

**Study design and participants**

The trial will be an unblinded, parallel-group, individually-randomised controlled trial to evaluate the superiority of an intensive 6-month education programme in comparison to the existing conventional 6-month education programme. The trial will run from 01/09/2013 to 30/10/2014, with all participants recruited from the endocrinology clinic at the UCTH in Cross River State, Nigeria between 1/09/2013 and 31/05/2014. Participants with type 1 or type 2 diabetes who are treated in the endocrinology clinic will be screened to determine their eligibility for the study. Eligible participants will be classed as those aged 18 years or older who have glycosylated haemoglobin (HbA_1c_ %) levels >8.5% (as measured at a maximum of 30 days prior to randomisation), are able to exercise, and can participate in the study if randomised. Participants will be excluded if they have significant eye disease limiting visual acuity, or lower extremity amputation limiting exercise.

After being given an explanation of the study, participants who meet the inclusion criteria will be asked to complete a written informed consent document. They will thereafter be invited to participate by a research assistant, who will handle the assignment of participants to study arms based on randomisation lists generated by a statistician. Lists will be kept in sealed, opaque envelopes until a participant has provided written informed consent and all baseline data has been collected.

**Procedures**

A nurse practitioner will coordinate the intervention. Participants’ HbA_1c_ (%) levels will be measured by obtaining 4μl of blood in a capillary tip, taken from a finger prick, and analysing the samples in a Clover A1c Analyser™ (EuroMedix). HbA_1c_ levels will be measured for all participants at baseline prior to randomisation, and again after 6-months exposure to either the conventional or intensive education programme. The outcome measure for the study will be defined as the change in HbA_1c_ (%) from baseline to 6-month follow-up.

Participants randomised to the intensive education arm will be exposed to a total of 12 structured teaching sessions lasting around 2 hours each, which will be held fortnightly over a 6-month period. These teaching sessions will be handled by certified diabetes educators with doctors handling the initial 6 sessions and nurses handling the remaining 6 sessions. Each session will comprise of lectures and group discussions centred around the core educational elements recommended by the International Diabetes Federation and reflected in the educational guide of the COMDIS-HSD group in the United Kingdom. The themes to cover are 1) diet and nutrition, 2) compliance with medications and the mechanism of medication action 3) exercise, 4) foot and skin care, 5) self-monitoring of glucose levels 6) smoking cessation and 7) blood pressure and cholesterol monitoring.

The sessions will be interactive and incorporate video sessions with educational materials to reinforce the desired lifestyle modifications. Adherence to these intervention sessions will be assessed by recording attendance at these sessions. Care will be taken to provide this education at a location that is well removed from the endocrinology clinic area to limit interactions between participants in each arm of the study (Unical Medical School Lecture Theatre). Mobile phone messages will also used regularly to remind participants to attend the educational sessions in a bid to limit the losses to follow up. There will be no tailoring or modification of the intervention throughout the course of this trial.

Participants in the conventional education group will go through the normal educational programmes offered to persons with diabetes in the endocrinology clinic, provided by trained diabetes educators and doctors. Before the start of any clinic, the diabetes educators usually engage participants in a group education session which lasts between 30 and 45 minutes. Principles of diabetes care such as nutrition, compliance with medications, foot and skin care are usually covered in these sessions. The meetings will take place in a room close to the outpatient clinics. After these sessions, the participants will go on to attend their clinic appointment.

Participants in both groups will be asked to return at 6-months after randomisation for measurement of HbA_1c_ levels by nurse practitioners, who will be blinded to the allocation of participants.

**Ethical considerations**

High ethical standards will be maintained throughout the entire duration of the research. Participation will voluntary and the cost of any tests conducted will not be borne by the participants. Participants will be allowed to withdraw from the study at any time they feel like and refusal to participate will have no untoward consequences. The result of the tests will be communicated to all participants

**Sample size, randomisation and blinding**

118 individuals will be tested. Participants will be randomised in a 1:1 allocation ratio using a randomisation list produced by a statistician, and based on a permuted block design using a computer random number generator, with a fixed block size of 4.

**Statistical analyses**

The primary analysis will be conducted on the intention-to-treat population (ITT) population, which will include all participants randomised into the study, and analyse participants according to the intervention group to which they were originally assigned. The following variables will be used in the multiple imputation model: change in HbA_1c_ (%) from baseline to 6-months, treatment group, baseline HbA_1c_ (%) level, sex and age. A sensitivity analysis will also be conducted based on the ITT population.

**Data management**

Data will be held in secure computers and anonymity will be maintained. The trial data will be destroyed after a period of three years**.**

**Composition of the teams**

Team 1

1. Dr Okon Essien – Principal Investigator
2. Dr Ofem Enang
3. Dr Boniface Stevens
4. Felicia Sunday – nurse
5. Charity Williams – nurse
6. Fiona Effiong - nurse

Team 2

1. Dr Akaninyene Otu
2. Dr Victor Umoh
3. Dr Crystal Effiong
4. Nse Umoh – nurse
5. Joy Omini – nurse
6. Blessing Etuk – nurse

Nurse practitioners

1. Monday Johnson
2. Justina Otu

Research assistants

1. Augustine Edem
2. Patience Duke

Statistician

Joseph Paul Hicks

**References**

1. Silva AA, Bosco DD. An educational program for insulin self-adjustment associated with structured self-monitoring of blood glucose significantly improves glycaemic control in patients with type 2 diabetes mellitus after 12 weeks: a randomized controlled pilot study. Diabetology & Metabolic Syndrome 2015, 7:2
2. International Diabetes Federation. IDF Diabetes Atlas 6^th^ edition. 2013. Accessed online on 10^th^ June 2015. Available at: [www.idf.org/diabetesatlas](http://www.idf.org/diabetesatlas).
3. Mendes AB, Fittipaldi JA, Neves RC, Chacra AR, Moreira Jr ED. Prevalence and correlates of inadequate glycaemic control: results from a nationwide survey in 6,671 adults with diabetes in Brazil. Acta Diabetol. 2010;47:137–45.
4. Ceriello A, Barkai L, Christiansen JS, Czupryniak L, Gomis R, Harno K, et al. Diabetes as a case study of chronic disease management with a personalized approach: the role of a structured feedback loop. Diabetes Res Clin Pract. 2012;98:5–10.
5. Parkin CG, Buskirk A, Hinnen DA, Axel-Schweitzer M. Results that matter: structured vs. unstructured self-monitoring of blood glucose in type 2 diabetes. Diabetes Res Clin Pract. 2012;97:6–15.
6. [Saudek](http://www.ncbi.nlm.nih.gov/pubmed/?term=Saudek%20CD%5Bauth%5D) CD, [Brick](http://www.ncbi.nlm.nih.gov/pubmed/?term=Brick%20JC%5Bauth%5D) JC. The Clinical Use of Hemoglobin A1c. J Diabetes Sci Technol. 2009 Jul; 3(4): 629–634.
7. Miller LV, Goldstein J. L'enseignement au malade sur sa maladie et son traitement, un succès thérapeutique, un échec du corps médical. Journ Annu Diabetol Hotel Dieu 1984; 193-207.
8. Annaswamy R, Helen G, Judith OB, Patricia M, Paul RC. A randomized trial comparing intensive and passive education in patients with diabetes mellitus. Arch Intern Med. 2002;162:1301-1304.

| Research Timetable | | | | | | | | | | | | | |  |
| --- | --- | --- | --- | --- | --- | --- | --- | --- | --- | --- | --- | --- | --- | --- |
| Tasks | Months 2013/2014 | | | | | | | | | | | | |  |
|  | Sept | Oct | Nov | Dec | Jan | Feb | Mar | Apr | May | Jun | July | Aug | Sep | Oct |
| Recruitment of team members |  |  |  |  |  |  |  |  |  |  |  |  |  |  |
| Development of data collection instrument |  |  |  |  |  |  |  |  |  |  |  |  |  |  |
| Training of research team |  |  |  |  |  |  |  |  |  |  |  |  |  |  |
| Pretesting of data collection instrument |  |  |  |  |  |  |  |  |  |  |  |  |  |  |
| Recruitment of participants |  |  |  |  |  |  |  |  |  |  |  |  |  |  |
| Intensive education and follow up |  |  |  |  |  |  |  |  |  |  |  |  |  |  |
| Progress reports |  |  |  |  |  |  |  |  |  |  |  |  |  |  |
| Collation of results |  |  |  |  |  |  |  |  |  |  |  |  |  |  |
| Data entry |  |  |  |  |  |  |  |  |  |  |  |  |  |  |
| Data analysis |  |  |  |  |  |  |  |  |  |  |  |  |  |  |
| Report writing |  |  |  |  |  |  |  |  |  |  |  |  |  |  |
| Distribution of report to stakeholders |  |  |  |  |  |  |  |  |  |  |  |  |  |  |
