# Supplementary material for: Intensive Patient Education Improves Glycaemic Control in Diabetes Compared to Conventional Education: A Randomised Controlled Trial in a Nigerian Tertiary Care Hospital
Source: PLoS One. 2017 Jan 3;12(1):e0168835. doi: 10.1371/journal.pone.0168835 (PMC5207750; doi:10.1371/journal.pone.0168835)
Supplement: S3 Text — (DOCX) [file pone.0168835.s003.docx]

**S3 Text Multiple Imputation Details.**

**Methodological details of the multiple imputation process used in the missing data sensitivity analyses.**

Overall 14 (11.9%) patients were lost to follow-up, 6 (10.2%) from the intensive education group and 8 (13.6%) from the conventional education group. The primary analysis assumed that missing outcome data (HbA_1c_ % measures at six months follow-up) were missing completely at random, following informal exploration that indicated no clear patterns to the missingness. Multiple imputation was therefore used in sensitivity analyses to deal with the missing outcome data, while assuming that the missing data were actually missing at random [1]. This was done using the PROC MI function in SAS (SAS 9.3, SAS Institute Inc), using the Markov Chain Monte Carlo algorithm option, and assuming a multivariate normal distribution for the missing outcome data [2].

The following variables were used in the multiple imputation model for both the crude and adjusted results: HbA_1c_ (%) at six month follow-up, treatment group, baseline HbA_1c_ (%) level, sex, age, education level, type of diabetes, years since diabetes diagnosis and waist circumference. All were considered potentially influential on the outcome, and were therefore included, but no suitable auxiliary variables were identified from the available dataset. 50 datasets were imputed and analysed with multivariate regression models using PROC MIXED to compare HbA_1c_ (%) at six month follow-up between participants allocated to the intensive education group and the conventional education group, whilst controlling for other explanatory variables (for the crude results the model only included baseline HbA_1c_ (%) level in addition to treatment group, but for the fully adjusted results the model included baseline HbA_1c_ (%) level, sex, age, education level, type of diabetes, years since diabetes diagnosis and waist circumference). The results of these analyses were then combined using PROC MIANALYZE to produce the final results (S6 Table).

**References**

1. Groenwold RHH, Donders ART, Roes KCB, Harrell FE, Jr., Moons KGM: Dealing With Missing Outcome Data in Randomized Trials and Observational Studies. *American Journal of Epidemiology* 2012, 175(3):210-217.

2. White IR, Royston P, Wood AM: Multiple imputation using chained equations: Issues and guidance for practice. *Statistics in Medicine* 2011, 30(4):377-399.
