# Supplementary material for: Intensive Patient Education Improves Glycaemic Control in Diabetes Compared to Conventional Education: A Randomised Controlled Trial in a Nigerian Tertiary Care Hospital
Source: PLoS One. 2017 Jan 3;12(1):e0168835. doi: 10.1371/journal.pone.0168835 (PMC5207750; doi:10.1371/journal.pone.0168835)
Supplement: S1 Table — (DOC) [file pone.0168835.s004.doc]

**Items to include when reporting a randomized trial in a journal or conference abstract**

| **Item** | **Description** | **Reported on line number** |
| --- | --- | --- |
| Title | Identification of the study as randomized | 38 |
| Authors * | Contact details for the corresponding author | See contact details |
| Trial design | Description of the trial design (e.g. parallel, cluster, non-inferiority) | 38 |
| Methods |  |  |
| Participants | Eligibility criteria for participants and the settings where the data were collected | 41-42 |
| Interventions | Interventions intended for each group | 39-41 |
| Objective | Specific objective or hypothesis | 39-41 |
| Outcome | Clearly defined primary outcome for this report | 44 |
| Randomization | How participants were allocated to interventions | 38 & 42-43 |
| Blinding (masking) | Whether or not participants, care givers, and those assessing the outcomes were blinded to group assignment | 38 |
| Results |  |  |
| Numbers randomized | Number of participants randomized to each group | 46 |
| Recruitment | Trial status | NA |
| Numbers analysed | Number of participants analysed in each group | 46-47 |
| Outcome | For the primary outcome, a result for each group and the estimated effect size and its precision | 48-50 |
| Harms | Important adverse events or side effects | NA |
| Conclusions | General interpretation of the results | 55-58 |
| Trial registration | Registration number and name of trial register | 58-59 |
| Funding | Source of funding | See funding statement |

**this item is specific to conference abstracts*
