# Supplementary material for: Intensive Patient Education Improves Glycaemic Control in Diabetes Compared to Conventional Education: A Randomised Controlled Trial in a Nigerian Tertiary Care Hospital
Source: PLoS One. 2017 Jan 3;12(1):e0168835. doi: 10.1371/journal.pone.0168835 (PMC5207750; doi:10.1371/journal.pone.0168835)
Supplement: S2 Table — (DOCX) [file pone.0168835.s005.docx]

**Table A. Multiple regression results for HbA_1c_ outcomes at six-month follow-up for all recruited participants in the intensive and conventional education groups with missing outcome data imputed using a multiple imputation or baseline observation carried-forward approach.**

|  | **Intervention-control mean difference estimate (95% CI); P-value*** | |
| --- | --- | --- |
|  | **Crude** | **Adjusted** |
| MI HbA_1c_ % | -1.8 (-1.1 to -2.4); <0.001 | -1.8 (-1.1 to -2.4); <0.001 |
| BOCF HbA_1c_ % | -1.6 (-2.2 to -1.1); <0.001 | -1.6 (-2.2 to -1.1); <0.001 |

* Intervention effect = estimated mean difference in HbA_1c_ % between intervention and control. Crude HbA_1c_ % estimates based on multivariate linear regression models, only adjusting for baseline HbA_1c_ (%). Adjusted HbA_1c_ % estimates based on multivariate linear regression models, adjusting for baseline HbA_1c_ (%), age, sex, diabetes type, education level, years since diabetes diagnosis and waist circumference.

MI HbA_1c_ % = results from multiply imputed analysis (see S5 Text for details), and BOCF HbA_1c_ % = results from baseline observation carried-forward approach analysis.

**Table B. Multiple regression results for HbA_1c_ and glycaemic control outcomes at six-month follow-up for type 1 and type 2 diabetes participants in the intensive and conventional education groups.**

|  | **Mean (SD) or n (%)** | | **Intervention effect estimate (95% CI); P-value*** | |
| --- | --- | --- | --- | --- |
|  | **Intensive education group (N = 53)** | **Conventional education group (N = 51)** | **Crude** | **Adjusted** |
| T1: n | 9 (17%) | 6 (11.3%) |  |  |
| T1: HbA_1c_ % | 8.3 (1.6) | 10.8 (1.9) | -2.8 (-4.4 to -1.2); <0.001 | -3 (-4.7 to -1.4); <0.001 |
| T1: Glycaemic control | 3 (33.3%) | 0 (0%) | ^a^ | ^a^ |
| T2: n | 44 (86.3%) | 45 (88.2%) |  |  |
| T2: HbA_1c_ % | 8.7 (1.9) | 9.9 (2) | -1.6 (-2.3 to -0.9); <0.001 | -1.6 (-2.3 to -0.9); <0.001 |
| T2: Glycaemic control | 16 (36.4%) | 7 (15.6%) | 4.3 (1.4 to 12.9); 0.009 | 6.3 (1.8 to 22.2); 0.005 |

* Intervention effects = estimated mean difference in HbA_1c_ % between the intensive and conventional education group, and estimated odds ratio of glycaemic control in intensive vs conventional education group.

Glycaemic control = HbA_1c_ <7%.

Crude HbA_1c_ % and glycaemic control estimates based on multivariate linear/logistic regression models of complete cases, only adjusting for baseline HbA_1c_ (%). Adjusted HbA_1c_ % and glycaemic control estimates based on multivariate linear/logistic regression models of complete cases, adjusting for baseline HbA_1c_ (%), age, sex, diabetes type, education level, years since diabetes diagnosis and waist circumference.

^a^ It was not possible to obtain meaningful crude or adjusted results for glycaemic control outcomes for type 1 diabetes participants due to the highly restricted sample size preventing models from estimating standard errors.

**Table C. Logistic regression analysis of possible correlates of missingness in HbA_1c_ outcome data.**

|  | **OR estimate (95% CI); P-value** | |
| --- | --- | --- |
|  | **Univariate** | **Multivariate** |
| Intensive education group (Ref = conventional education group) | 0.7 (0.2 to 2.4); 0.6 | 0.7 (0.2 to 2.2); 0.6 |
| Baseline HbA_1c_ % | 0.8 (0.57 to 1.2); 0.4 | 0.9 (0.6 to 1.2); 0.4 |
| Type 2 diabetes (Ref = type 1) | 0.8 (0.12 to 5.1); 0.8 | 1 (0.2 to 5); 0.99 |
| Sex (Ref = male) | 0.8 (0.2 to 2.8); 0.7 | 0.9 (0.3 to 2.7); 0.8 |
| Age | 1 (0.9 to 1.1); 0.9 | 1 (0.9 to 1.1); 0.8 |
| Waist circumference | 0.98 0.9 to 1.1); 0.6 | 1 (0.9 to 1); 0.4 |
| Secondary education (Ref = none/primary) | 2.22 (0.5 to 10); 0.3 | 2.1 (0.5 to 9); 0.3 |
| Tertiary education (Ref = none/primary) | 0.99 (0.2 to 5.1); 0.99 | 0.9 (0.2 to 4.3); 0.9 |

Univariate OR results are from logistic regression models estimating the relationship between the relevant independent variable and a dummy binary variable for missingness in the outcome data. Multivariate OR (i.e. adjusted OR) results are from a logistic regression model estimating the relationships between all listed independent variables and a dummy binary variable for missingness in the outcome data.
